# On the amyloid datasets used for training PAFIG ­ how (not) to extend the experimental dataset of hexapeptides

**DOI:** 10.1186/1471-2105-14-351

**Published:** 2013-12-04

**Authors:** Malgorzata Kotulska, Olgierd Unold

**Affiliations:** 1 Institute of Biomedical Engineering and Instrumentation, Wroclaw University of Technology, 50-370 Wroclaw, Poland; 2 Institute of Computer Engineering, Control and Robotics, Wroclaw University of Technology, 50-370 Wroclaw, Poland

**Keywords:** Machine learning, Amyloid, Intramolecular contact sites, Hot spot

## Abstract

**Background:**

Amyloids are proteins capable of forming aberrant intramolecular contact sites, characteristic of beta zipper configuration. Amyloids can underlie serious health conditions, e.g. Alzheimer’s or Parkinson’s diseases. It has been proposed that short segments of amino acids can be responsible for protein amyloidogenicity, but no more than two hundred such hexapeptides have been experimentally found. The authors of the computational tool Pafig published in BMC Bioinformatics a method for extending the amyloid hexapeptide dataset that could be used for training and testing models. They assumed that all hexapeptides belonging to an amyloid protein can be regarded as amylopositive, while those from proteins never reported as amyloid are always amylonegative. Here we show why the above described method of extending datasets is wrong and discuss the reasons why the incorrect data could lead to falsely correct classification.

**Results:**

The amyloid classification of hexapeptides by Pafig was confronted with the classification results from different state of the art computational methods and the outputs of all methods were studied by clustering analysis. The clustering methods show that Pafig is an outlier with regard to other approaches. Our study of the statistical patterns of its training and testing datasets showed a strong bias towards STVIIE hexapeptide in their positive part. Different statistical patterns of seemingly amylo -positive and -negative hexapeptides allow for a repeatable classification, which is not related to amyloid propensity of the hexapetides.

**Conclusions:**

Our study on recognition of amyloid hexapeptides showed that occurrence of incidental patterns in wrongly selected datasets can produce falsely correct results of classification. The assumption that all hexapeptides belonging to amyloid protein can be regarded as amylopositive and those from proteins never reported as amyloid are always amylonegative is not supported by any other computational method. This is in line with experimental observations that amyloid propensity of a full protein can result from only one amyloidogenic fragment in this protein, while the occurrence of amyliodogenic part that is well hidden inside the protein may never lead to fibril formation. This leads to the conclusion that Pafig does not provide correct classification with regard to amyloidogenicity.

## Background

Amyloids are proteins capable of forming aberrant intramolecular contact sites that are characteristic of the beta zipper configuration, and can lead to fibrils instead of the functional structure of a protein [1-5]. The processes of amyloid oligomerization, which precedes fibril formation is currently regarded as responsible for serious health conditions, such as Alzheimer’s disease (amyloid-β, tau), Parkinson’s disease (α-synuclein), type 2 diabetes (amylin), Creutzfeldt-Jakob’s disease (prion protein), Huntington disease (huntington), amyotrophic lateral sclerosis (SOD1), and many others (for a review see e.g.) [6]. Therefore, it is of great interest to develop methods for predicting mechanisms leading to this phenomenon. It has been proposed that short segments of amino acids can be responsible for the amyloidogenic properties [7,8]. Those fragments are harmless only when they are buried inside a protein. The fragments responsible for amyloidogenicity of the whole protein are believed to be 4–10 residues long and it is often assumed that 6-residue fragments with amyloidogenic properties are sufficient “hot spots” [9]. Recognition of amyloidogenic fragments can be obtained by computational approach, for example physico-chemical methods, e.g. Tango [10], ZipperDB [9,11], Pasta [12], AggreScan [13], PreAmyl [14], Zyggregator [15], CamFold [16], NetCSSP [17], FoldAmyloid [18], AmyloidMutant [19,20], BetaScan [21], and consensus AmylPred [22]. Statistical methods have also been employed in the classification. In our previous work we used classical machine learning methods [23] based on WEKA [24]. Other methods include Waltz [25] using Position Specific Scoring Matrices (PSSM), or Bayessian classifier and weighted decision tree applied to long sequences of bacterial antibodies [26].

No more than two hundreds of such hexapeptides have been experimentally found. New computational algorithms are trained or validated on the scarce experimental dataset. Two papers published in BMC Bioinformatics, presenting machine learning methods - Pafig [27] and another approach based on Pafig [28], used their own method for extending the training and testing datasets. The authors assumed that all hexapeptides that belong to an amyloid protein can be regarded as amylo-positive, while those from proteins never reported as amyloid are always amylo-negative. Different machine learning methods were then applied to classify amyloid hexapeptides trained on a few thousand of full-length proteins cut into hexapeptides, which were labeled according to this scheme. The classification, validated on hexapeptides obtained in the same way, produced seemingly good results.

However, due to experimental observations, amyloid propensity of a full protein can result only from one amyloidogenic fragment in this protein, while the occurrence of amyloiodogenic part, which is well hidden inside the protein, may never lead to fibril formation. This was confirmed by results of 3D profile method [9], which produced the largest computational database of potential amyloid hexapeptides – ZipperDB [11]. In the database there are very many examples of proteins including highly amyloidogenic fragments that have never been observed to form an amyloid. It is possible that those fragments are screened inside the protein and deprived of contacts with other fragments of high amyloid propensity, hence unable to start oligomerization and fibril formation.

Therefore, we decided to look closer at the datasets proposed in Pafig (Hexpepset) and validate the results of this method, which was trained on a dataset obtained contrary to these observations. For this purpose we performed statistical analysis of the dataset with regard to possible false patterns or undesirable biases. Then we used other state of the art computational methods to classify amyloid hexapeptides and compare their results with Pafig by means of clustering approach. The objective was to study how compatible is Pafig to other classification methods.

## Results and discussion

### Dataset

**Figure 1:**
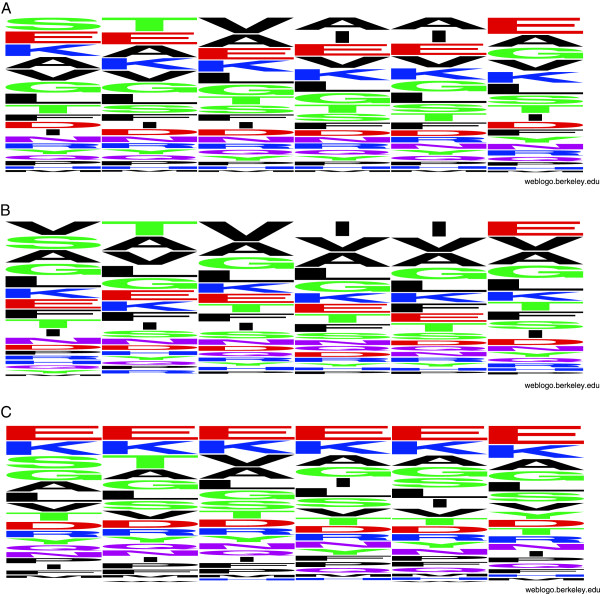
**Statistical distribution of aminoacids in hexapeptides from training Pafig dataset.** Graphical representation of the Hexpeptset logo: **(A)** all hexapeptides, **(B)** positive and **(C)** negative parts of the Hexpeptset.

The analysis of the total Hexpepset shows strong bias towards STVIIE hexapaptide, which can be observed at the Hexpepset logo (Figure 1), generated with WeBLogo [29]. This bias originates from the contribution of the largest up-to-date experimental amyloid hexapeptide dataset - AmylHex [9], which was incorporated into the Hexpeptset. In the positive part of the Hexpeptset, 66 hexapeptides (5.4%) come from AmylHex(+). Additionally, Hexpeptset(+) includes 13 incorrect hexapeptides (1%) that belong to AmylHex(−). Hexpeptset(−) does not include any hexapeptide from AmylHex. The influence of AmylHex on Hexpeptset is strong. The bias also means that the peptides are not representative of the protein world, which was the main criticism towards AmylHex dataset [10]. We tested the Hexpeptset with regard to its representativeness by comparing to UniProt statistics. Table 1 presents the ratio of each residue contained in Hexpeptset versus UniProt representation, which takes into account unequal contribution from different amino acids. Numbers greater than 1 indicate over-represented residues; STVIIE is presented in bold, the most abundant residues are in red.

**Table 1:** Statistical distribution of Pafig training dataset

**Residue position**	**1**	**2**	**3**	**4**	**5**	**6**
K	1.5	1.5	1.5	1.5	1.5	1.4
R	0.7	0.7	0.7	0.8	0.8	0.7
D	0.9	0.9	0.9	0.8	0.8	0.9
E	1.3	1.3	1.3	1.3	1.3	**1.8**
N	1.1	1.0	1.1	1.0	1.0	1.0
Q	0.9	0.9	1.0	1.0	0.9	0.9
P	0.7	0.7	0.6	0.7	0.6	0.6
H	1.0	1.1	1.1	1.0	1.1	1.0
M	0.8	0.7	0.7	0.7	0.8	0.7
C	0.5	0.6	0.6	0.6	0.6	0.5
S	**1.3**	0.8	0.8	0.8	0.9	0.9
T	1.1	**1.7**	1.0	1.0	1.0	1.0
F	1.2	1.2	1.2	1.2	1.2	1.1
W	0.9	1.0	0.9	0.9	0.9	0.8
Y	1.2	1.2	1.2	1.2	1.2	1.5
V	1.1	1.1	**1.6**	1.1	1.2	1.1
L	0.7	0.7	0.8	0.7	0.7	0.7
I	0.8	0.8	0.8	**1.4**	**1.4**	0.8
G	1.1	1.0	1.0	1.0	1.1	1.1
A	0.9	0.9	1.0	1.0	1.0	0.9

Statistical distribution of Pafig full training dataset, including all positive and negative hexapeptides, normalized versus frequencies of aminoacid occurrence in all proteins deposited in UniProt. The expected values for a well balanced training dataset should equal 1. The values above 1 denote over-representation of a residue at the specific location of training hexapeptides, values below 1 show under-representation. The bias from STVIIE is in bold.

The positive (Figure 1) and negative (Figure 1) parts of Hexpeptset exhibit patterns, which are different for positive and negative sets. This fact can be sufficient reason for the machine learning methods, trained and tested on this dataset, to be able to learn to distinguish hexapeptides from these two datasets. The question arises as to whether these patterns are related to amyloid propensity or did they appeared incidentally with strong contribution of AmylHex bias to positive Hexpeptset.

### Machine learning methods reveal two clusters

The Hexpeptset dataset, containing a binary classification of 2452 hexapeptides, was applied to three state of the art methods FoldAmylod [18], Waltz [25], and AmylPred [30]. The results of classification can be seen as a binary matrix (see Additional file 1). To identify similarity or dissimilarity between all examined methods (i.e. Pafig, FoldAmyloid, Waltz, and AmylPred) the clustering was applied (see Methods for details).

**Figure 2:**
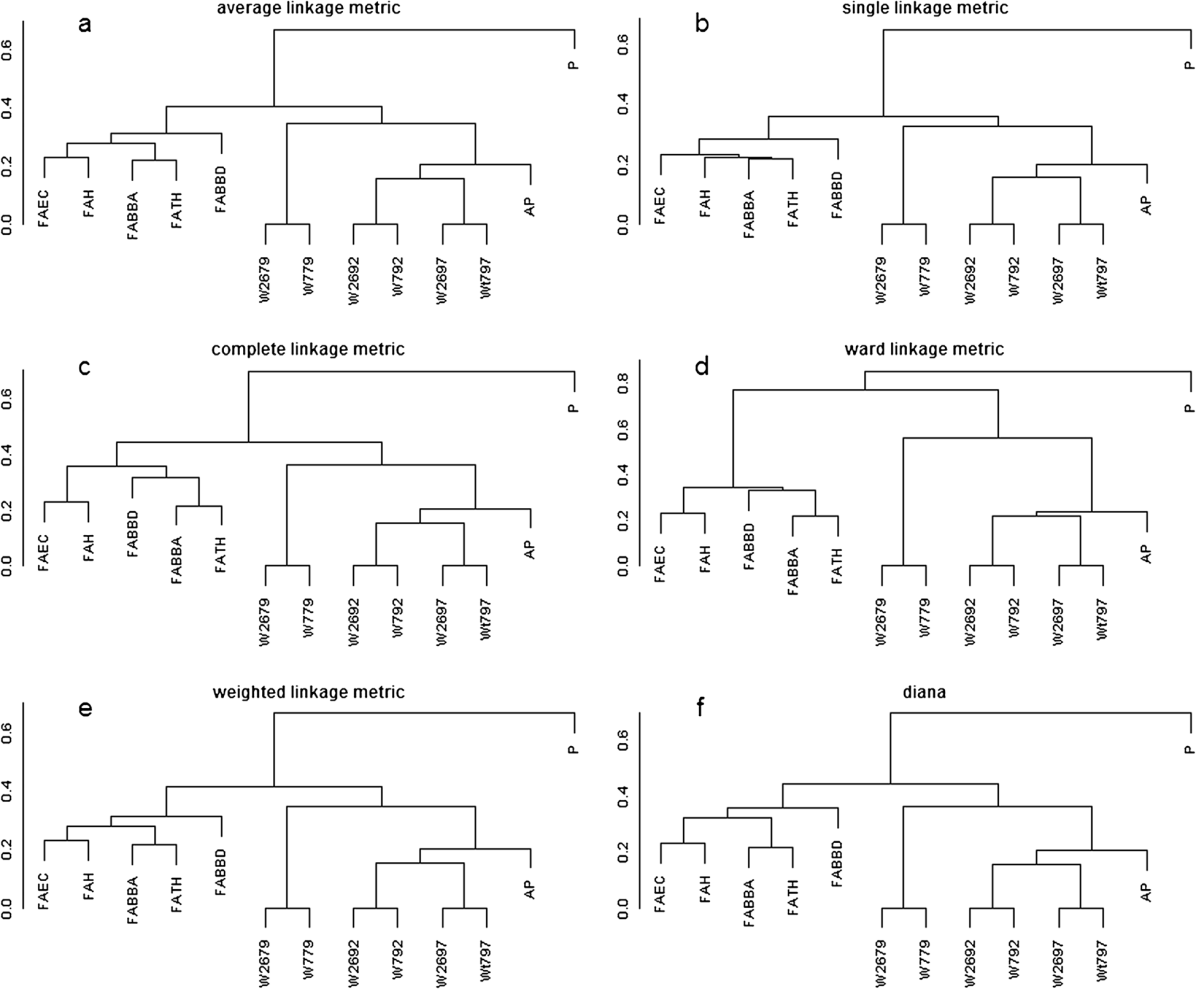
**Dendrograms of unsupervised hierarchical clustering for different linkage metrics and Sokal & Michener distance.** Dendrograms for Sokal & Michener distance and **(a)** average, **(b)** single, **(c)** complete, **(d)** ward, **(e)** weighted, and **(f)** diana linkages. Pafig method is placed in a separate cluster, regardless of distance metric and hierarchical clustering method.

The clusterSim package of R programming language, applied for testing all combinations of the number of clusters, distance metrics, and clustering methods, revealed two distinctly different groups of methods, i.e. FoldAmyloid, Waltz, and AmylPred located in one cluster, and Pafig in the other one. The Baker and Hubert index gained the highest possible value of 1. The exemplary dendrograms for different linkage metrics created by unsupervised hierarchical clustering (agnes and diana) are presented in Figure 2. All of them indicate two main clusters, in which Pafig is always located in a separate cluster. Interestingly, two distinct subgroups in the first cluster can be found: the first sub cluster consists of FoldAmyloid variants, whereas the second sub cluster is composed of Waltz variants and AmylPred. Since AmylPred is a consensus method incorporating several other methods, this can show greater similarity to Waltz approach.

**Figure 3:**
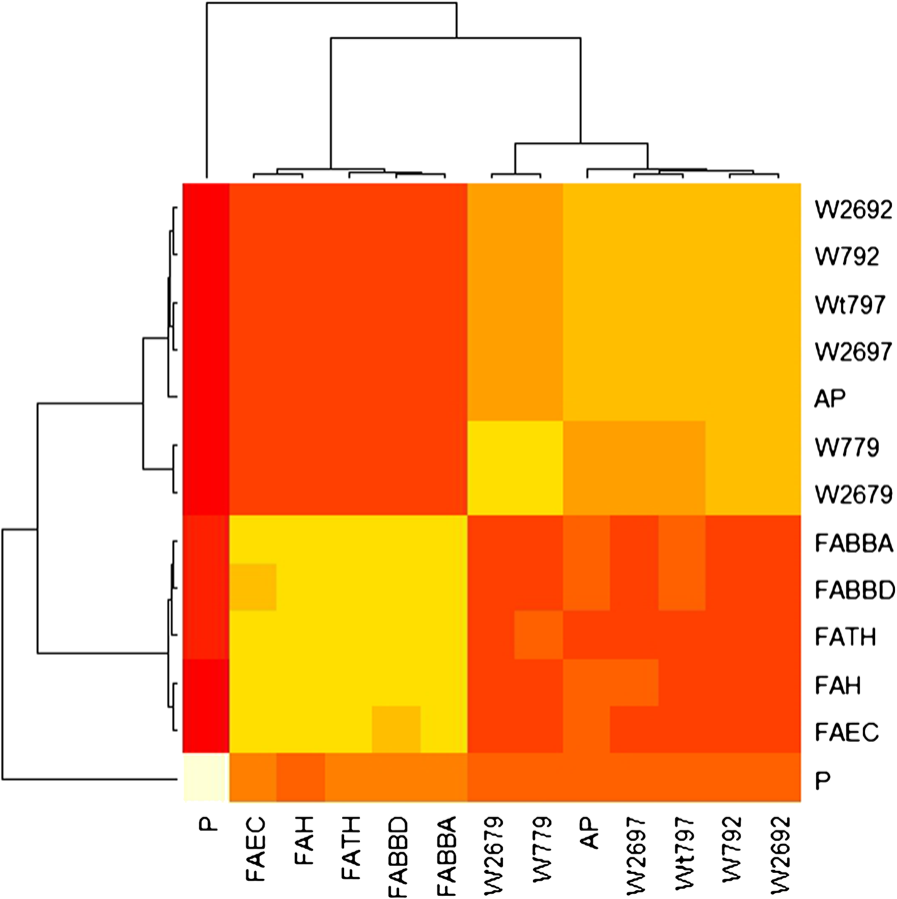
**Heatmap of merged consensus matrix for two clusters.** Heatmap showing the similarity within of the two groups of methods: Waltz (denoted by Wxxx, where, for example, Waltz at pH = 2.6 and threshold = 79, was denoted by W2679, and Waltz at pH = 7 and threshold = 79 was denoted as W779), AmylPred (AP), and FoldAmyloid (Fxxxx). Pafig (P) was clustered as a quite different approach. Similarity was calculated using merged distance matrix over different clustering algorithms (agnes, pam, hclust, kmeans, and diana).

To confirm the obtained results, the stability-based clustering method was applied. The merged consensus clustering, which used resampling of data and different clustering algorithms (agnes, k-means, pam, hclust, and diana), created the merged consensus matrix that was generated by unweighted averaging of the consensus matrices provided by each clustering algorithm. The merged matrix could be used as a distance matrix. Figure 3 presents the heatmap of merged consensus matrix calculated for two clusters. Bootstrapping of data and using a bunch of clustering algorithms reaffirmed the conclusions drawn above – all methods are divided into two branches, Waltz and AmylPred are recognized as sub-branch within one branch with FoldAmyloid, whereas Pafig is clustered within the other branch.

**Figure 4:**
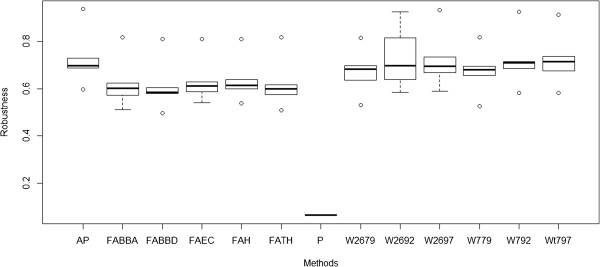
**Box plots showing the robustness values associated with the structures of each of the two cluster outcomes.** Each box plot represents the membership robustness values of a method over different consensus clustering algorithms (agnes, pam, hclust, k-means, and diana). The membership robustness is calculated as the average connectivity between the method and all of the other methods of the cluster. While consensus clustering produces more or less identical membership robustness values for all methods but for Pafig, the robustness for Pafig method is noticeably lower reflecting its heterogeneity.

Figure 4 shows a box plot with the robustness values associated with two clusters. From the Figure 4 it is clear, that the membership robustness values are noticeably lower, on average, for the Pafig method. Pafig results have to be treated as significantly different from the results gained by the other methods.

In the additional file (“Dataset of hexapeptides with amyloidogenic classification”) we compare the classification results of PAFIG and other classical computational methods used in this study. The file also includes the sheets called “non-amyloids” and “amyloids”, which compares the unanimous voting (“all agree”) of the classical methods over the dataset Hexpepset with the Pafig classification. The classical methods (except Pafig) are regarded as base learning algorithms in heterogeneous ensemble method with unanimous strategy. The results of this analysis show that hexapeptides regarded as non–amyloids by the unanimous voting (1648 instances) are identically recognized by Pafig in only 57%, which is close to random. On the other hand Pafig identically recognized 100% of hexapeptides classified as “amyloids”, however this set included only 28 instances.

## Conclusions

Our study on recognition of amyloid hexapeptides showed that occurrence of incidental patterns in wrongly selected datasets can produce falsely correct results of classification. In the Hexpeptset dataset, used for training machine learning methods of Pafig, “amyloid” part of data appeared strongly biased towards STVIIE hexapeptide, which comes from experimental AmylHex dataset. This bias contributed to the pattern, which could be recognized by machine learning. On the other hand, “non-amyloid” part of Hexpeptset lacks this bias, although it exhibits a different pattern of its hexapeptides, which is not position-dependent. The difference in patterns of these sets was sufficient for “successful” training of machine learning methods. However, this training did not allow for a correct classification of amyloid hexapeptides. Comparison of classification results between Pafig and other computational state of the art methods, by means of clustering methods, showed that Pafig is an outlier with regard to the classification results. This means that its classification is different and not related to amyloid properties of hexapeptides.

Our results of data analysis are in line with experimental observations – amyloid propensity of a full protein can result from only one amyloidogenic fragment in this protein, while the occurrence of amyliodogenic part that is well hidden inside the protein may never lead to fibril formation.

## Methods

### Dataset

The analysis was performed on Hexpepset dataset, introduced in Pafig. The Hexpepset dataset was downloaded from website of Pafig [31] and consisted of 2452 hexpeptides (1226 positive samples and 1226 negative samples). The positive samples in the Hexpepset dataset were collected by Pafig’s authors scanning proteins that are proved as fibrils forming with a six-residue window. The negative part contained samples obtained by scanning the proteins that had not been experimentally proved to form fibrils.

### Validation with other classification methods

To test a homogeneity of the Pafig dataset with other state of the art amyloid datasets, we used the Pafig Hexpeptdataset dataset, denoted by P, as an input for three methods: FoldAmyloid [18,32], Waltz [25,33], and AmylPred AP [30,34] (as of December 2012). All standard FoldAmyloid methods were applied: contacts – denoted by FAEC, bone-bone donors FABBD, bone-bone acceptors FABBA, hybrid (contacts + donors) FAH, and triple hybrid (contacts + donors + acceptors) FATH. Waltz was run with its standard optimizations for overall performance and sensitivity. The following notation was used: Waltz pH = 2.6 threshold 79 was denoted as W2679, Waltz pH = 7 threshold 79 - W779, Waltz pH = 2.6 threshold 92 - W2692, Waltz pH = 7 threshold 92 - W792, Waltz pH = 7 threshold 97 - W797, Waltz pH = 7 threshold 79 - W779. The objective was to calculate the similarity (dissimilarity) of different predictive models (i.e. Pafig, FoldAmylod, Waltz, AmylPred) over one dataset. To obtain this goal we used clustering techniques.

### Clustering of binary data

A binary matrix is used as data when clustering all binary classifications of 2452 hexapeptides taken from Pafig dataset over FoldAmyloid, Waltz and AmylPred methods (see Additional file 1). Up to now numerous binary similarity measures and distance measures have been used. In the survey by Choi et al. [35], 76 binary similarity and distance measures were collected for dichotomous data. We employ three different distance measures: Sokal & Michener [36]:

$$\mathrm{sSM}\;=\;\frac{\left(a+d\right)}{\left(a+b+c+d\right)}$$

Rogers & Tanimoto [37]:

$$\mathrm{sRT}=\;\frac{\left(a+d\right)}{a+2\;\left(b+c\right)+d)}$$

and Sokal & Sneath [38]:

$$\mathrm{sSS}\;=\;\frac{a\;d}{\sqrt{\left(a+b\right)\;\left(a+c\right)\;\left(d+c\right)}}$$

where *a*, *b*, *c*, *d* are the elements of the contingency table of binary data, N_2x2_, in which *a* = *n*_1,1_, *b* = n_1,0_, *c* = *n*_0,1_, and *d* = *n*_0,0_. Note that all mentioned above binary similarity measures take into account both positive (*n*_11_) and negative matches (*n*_00_). This is because it is important to reflect the same classification of a hexapeptide by examined methods.

To measure an internal cluster quality index and find the optimal number of clusters, we used Baker and Hubert clustering criterion [39] which is among the most effective ones [40,41]. Baker and Hubert index is an adaptation of Goodman & Kruskal’s Gamma statistics, and it is calculated as follows:

$$G\left(u\right)=\frac{s_{+}-s_{-}}{s_{+}+s_{-}},$$

where *s*_*+*_ is the number of concordant comparisons (the number of times that a pair of samples not clustered together have a larger separation than pairs that were in the same clusters), *s*_*−*_ is the number of discordant comparisons (within-cluster dissimilarity is strictly greater than a between-cluster dissimilarity), *u* is the number of clusters (*u* = 2,.., *n* −1), and *n* is the number of objects. The value of *u*, which maximizes *G*(*u*), is regarded as specifying the number of clusters.

The clusterSim package of the R programming language was employed to determine the proper cluster numbers [42]. To find the optimal value of an internal cluster quality index (Baker and Hubert index), and thereby the optimal number and content of clusters, the package varies all combinations of distance measures (Sokal & Michener, Rogers & Tanimoto, and Sokal & Sneath) and clustering methods (single link, complete link, average link, McQuitty, k-medoids, Ward, centroid, median). All these combinations are tested against different number of clusters (from 2 to 8).

To prove a reliability of the obtained results, i.e. the identification of the correct number of clusters, we used stability-based method for cluster validity. The stability-based methods are the most robust and best performing in terms of prediction [43]. Here, consensus clustering [44] extended to merge consensus clustering by Simpson [45] was chosen as a stability-based method of creating a robust cluster outcome. In consensus clustering multiple clustering algorithms are applied with a bootstrapping approach, i.e. sampling and clustering is repeated many times to find reliable cluster members. The obtained results are used to calculate cluster and membership robustness. Simpson et al. [45] extended this method to so called merged consensus clustering by applying many different clustering algorithms.

## Competing interests

The authors declare that they have no competing interests.

## Authors’ contributions

MK proposed the inconsistence of the dataset generation method with biological observations and performed the statistical data analysis. OU performed clustering analysis. Both authors wrote and approved the final manuscript.

## Supplementary Material

Additional file 1 **Dataset of hexapeptides with amyloidogenic classification.** It represents the classification results of the computational methods and the consensus result of all those methods (except Pafig) showing which hexapeptides seem improbable to form amyloids. The additional spreadsheets in the file (called “non-amyloids” and “amyloids”) include comparison of unanimous voting of the methods over the dataset Hexpepset with Pafig classification“. Click here for file
